# High Prevalence of Undernutrition among Children in Gondar Town, Northwest Ethiopia: A Community-Based Cross-Sectional Study

**DOI:** 10.1155/2017/5367070

**Published:** 2017-12-12

**Authors:** Zegeye Abebe, Degefaye Zelalem Anlay, Belete Biadgo, Asemarie Kebede, Tsedalu Melku, Bamlaku Enawgaw, Mulugeta Melku

**Affiliations:** ^1^Department of Human Nutrition, Institute of Public Health, College of Medicine and Health Sciences, University of Gondar, P.O. Box 196, Gondar, Ethiopia; ^2^Department of Community Health Nursing, School of Nursing, College of Medicine and Health Sciences, University of Gondar, Gondar, Ethiopia; ^3^Department of Clinical Chemistry, School of Biomedical and Laboratory Sciences, College of Medicine and Health Sciences, University of Gondar, P.O. Box 196, Gondar, Ethiopia; ^4^School of Nursing, College of Medicine and Health Sciences, University of Gondar, Gondar, Ethiopia; ^5^School of Medicine, College of Medicine and Health Sciences, University of Gondar, Gondar, Ethiopia; ^6^Department of Hematology and Immunohematology, School of Biomedical and Laboratory Sciences, College of Medicine and Health Sciences, University of Gondar, P.O. Box 196, Gondar, Ethiopia

## Abstract

**Objective:**

To assess undernutrition and associated factors among children aged 6–59 months in Gondar Town, northwest Ethiopia.

**Methods:**

A community-based cross-sectional study was conducted in 2014. Multistage sampling method was used to select study participants. Structured interviewer administered questionnaire and anthropometric measurements were used. Binary logistic regression was fitted to identify associated factors.

**Results:**

The prevalences of wasting and stunting were 6.8% and 45.7%, respectively. Higher odds of wasting were observed among children whose fathers were daily laborers (AOR = 2.63), children who had eating problem (AOR = 2.96), and those who were not exclusively breast-fed for the first six months (AOR = 5.63). Similarly, higher odds of stunting were found among female children (AOR = 1.65), children who lived in households having four to six families (AOR = 2.14), and children who did not start breast-feeding within one hour of birth (AOR = 0.67).

**Conclusion:**

Childhood undernutrition was a significant problem. Child eating problem, paternal occupation, and exclusive breast-feeding were associated with wasting, whereas family size, child sex, and breast-feeding initiation time were associated with stunting. Therefore, strengthening of early initiation and exclusive breast-feeding, promoting healthcare seeking behavior, and designing social support programme for poor family are recommended to reduce undernutrition.

## 1. Background

Undernutrition encompasses all stunting, wasting, underweight, and micronutrient deficiencies [1, 2]. According to World Health Organization (WHO), undernutrition is defined as *Z*-scores less than −2 standard deviations, irrespective of the indicators used [3]. Child undernutrition (stunting and wasting) has been a serious global public health problem for the past many decades, especially in the developing countries including Ethiopia [4, 5]. It is considered as a very critical public health problem when the prevalence of stunting and that of wasting among children are higher than 40 and 15%, respectively [6]. The consequences of undernutrition are numerous, such as increased mortality, irreversible brain damage, and negative effect on cognitive ability, which have a cumulative effect on social development [7–9]. In addition, it has been associated with adverse functional consequences, such as overweight, obesity, insulin resistance, hypertension, dyslipidemia, a reduced capacity for manual work, and other chronic noncommunicable diseases during adolescence and adulthood [8, 10, 11]. Furthermore, it has a negative effect on future reproductive outcomes [12].

Globally, around 8 and 26% of children below five years of age are wasted and stunted, respectively. The highest prevalence of childhood undernutrition is found in Africa (38.2%) followed by Southeast Asia (27.6%), while the lowest magnitude is found in Latin America and the Caribbean countries (13.5%) [13, 14].

In Ethiopia, the magnitude of childhood undernutrition has decreased from 58% in 2000 to 40% in 2014 with regional differences ranging from 52% in Amhara to 22% in Addis Ababa [15, 16], but it is sustained as the major public health problem. For instance, 28% of all child mortality, 16% of all repetitions in primary school, and 8% of the workforce lost are associated with undernutrition. In addition, it is also associated with an estimated annual cost loss of 55.5 billion Ethiopian Birr (ETB), which is equivalent to 16.5% of the country's gross domestic product (GDP) [17].

Previous researchers from different corners of the globe confirmed that undernutrition is a complex interaction between different factors. Accordingly, poor child feeding practice [18], being male [19], poor household wealth status [19], prelacteal feeding and large household size [4, 5], and poor paternal educational status and an eating problem [5] were factors associated with undernutrition.

The Government of Ethiopia has planned to reach the zero-level undernutrition by 2030 [20]. Accordingly, it endorsed a National Nutrition Program, prepared infant and young child feeding manual, implemented monthly child growth and monitoring program, and has been working in collaboration with different nongovernmental organizations, such as Micronutrient Initiatives (MI), United Nations Children's Fund (UNICEF), and Save the Children. However, about 40 and 9% of children below five years of age were stunted and wasted, respectively [16].

In addition, level of undernutrition depends on the ecological setting of the country, and there is a need to investigate the magnitude and risk factors to generate comprehensive data for policy makers and governmental and nongovernmental organizations working on child health and nutrition. Therefore, the aim of this study was to assess the magnitude and associated factors of undernutrition among children aged 6–59 months in Gondar Town, northwest Ethiopia.

## 2. Materials and Methods

### 2.1. Study Period and Setting

The study was conducted in Gondar Town from 15 October to 30 November 2014. The town is found in North Gondar Zone, Amhara Regional State of Ethiopia, and is located at 750 km from Addis Ababa to the northwest. According to population projection rate, a population of around 300,000 had been estimated to have resided in Gondar Town in 2014. Administratively, the town is divided into 12 administrative areas (subcities) which consist of 21 kebeles* (the smallest administrative units in Ethiopia)*.

### 2.2. Study Participants, Sample Size, and Sampling Procedure

All children who are aged 6–59 months and had lived in Gondar Town for at least six months were eligible for the study. The sample size of the study was calculated using the OpenEpi software, version 2.3, by assuming 46% of stunting prevalence [4], a 95% Confidence Interval, and a 5% margin of error. Finally, the sample size of 764 was obtained by considering the design effect of 2. A multistage sampling followed by the simple random sampling technique was employed to reach the study participants. Initially, four of the 12 subcities were selected by the lottery method. Then, four* kebeles* (one kebele from each subcity) were selected. A total number of children in the selected* kebeles *were obtained from health extension workers, and then the total number of children included in the study was proportionally allocated to each* kebele*. Finally, a simple random sampling method was used to select the study subjects from the list of children in each* kebele*.

### 2.3. Data Collection Instrument and Procedure

A structured interviewer-administered questionnaire was used to collect data. The questionnaire was adopted from EDHS (Ethiopian Demographic and Health survey) 2011 and other similar studies with some modifications to fit the local context. The questionnaire consisted of the sociodemographic characteristics of the children and their parents, child health, dietary practice related factors, and maternal health service utilization.

The questionnaire was initially prepared in English and translated to Amharic and, finally, retranslated to English to maintain consistency. A pretest was done on 5% of the sample outside the study area. Two-day training was given to data collectors and supervisors. The data were collected by clinical nurses and public health experts.

### 2.4. Assessment of Comorbidity Symptoms and Eating Problem

Assessment of comorbidity symptoms was begun by asking the mothers using a two-week recall period. Finally, the prevalence of undernutrition was compared among children who had the symptoms and those who did not. Similarly, eating problem was assessed by asking the mothers whether or not the child faced difficulty of swallowing, eating things that are not really food, and eating only certain types of food.

### 2.5. Assessment of Exclusive Breast-Feeding

Children were considered exclusively breast-fed for the first six month when the child had only received breast milk (including breast-feeding by a wet nurse and feeding expressed breast milk and children received ORS, drops, and syrups) [21].

### 2.6. Anthropometric Measurement

Birth date was determined by asking mothers and by cross-checking against immunization status certificates of the children. The weight of children aged 24–59 months was taken in light clothes and no shoe to the nearest 0.1 kg by a Seca beam balance (Germany, serial number 5755107131646 with graduation of 0.1 kg and measuring up to 160 kg). Similarly, the weight of children aged 6–23 months was measured by the salter scale to the nearest 0.1 kg (Germany, serial number 3541317009 with graduation of 0.1 kg and measuring up to 20 kg). The height of children aged 24–59 months was measured in Frankfurt (the child's head, shoulders, buttocks, knees, and heels touch the vertical board) position using the Seca vertical height scale (Germany, model number 213) to the nearest 0.1 cm, whereas the length of children aged 6–23 months was taken by a measuring board to the nearest 0.1 cm in recumbent position. Instrument calibration was done before measuring the next child. The weight and height of mothers were also measured in standing position using Seca beam balance and Seca vertical height scale, respectively.

The anthropometric data of the children were entered into the ENA/SMART software, version 2011. *Z*-scores of nutritional indices, such as Weight-for-Age (WAZ), Weight-for-Height (WHZ), and Height-for-Age (HAZ), were calculated using the WHO Multicenter Growth Reference Standard. Finally, children were classified as stunted, underweight, and wasted when the HAZ, WAZ, and WHZ scores were less than −2 standard deviations (SD), respectively [3]. Maternal body mass index (BMI) was calculated.

### 2.7. Data Processing and Analysis

The collected data were manually checked for completeness and consistency of responses. Then, the data were entered into Epi Info, version 7, and exported to SPSS, version 20, for further analysis. Descriptive statistics were used to summarize variables. Both bivariable and multivariable binary logistic regression analyses were used to identify variables associated with undernutrition. Variables with a *p* value less than 0.2 in the bivariable analysis were fitted into the multivariable binary logistic regression analysis to control the possible effect of confounding. Both Crude Odds Ratio (COR) and Adjusted Odds Ratio (AOR) with the corresponding 95% Confidence Interval (CI) were calculated to show the strength of association. Finally, in the multivariable analysis, variables with a *p* value less than 0.05 were considered statistically significant.

## 3. Results

### 3.1. Sociodemographic and Socioeconomic Characteristics of Parents

A total of 707 child-mother pairs were included in the study. The median age of the mothers was 27 years (Interquartile Range (IQR): 25–31 years). About 9.6% of the respondents had a family size of greater than seven. Around one-third (31.1%) of the mothers were unable to read and write. Most (70.7%) and nearly one-third (32.9%) of the mothers and their husbands were housewives and government employees, respectively (Table 1).

### 3.2. Child Feeding and Health-Related Characteristics

Of the total children, 378 (53.5%) were males. The mean (±SD) age of the children was 30 ± 14.39 months. One-fifth (21.5%) of the children were sick two weeks prior to the date of survey. Four (0.6%) of the children had vitamin A deficiency (Bitot's spot) (Table 2). Fifty-eight (8.2%) of the children were given prelacteal feeding, and 5.8% of the mothers used bottles for complementary feeding. More than two-thirds (81.6%) of children were exclusively breast-fed for the first six months (Table 3).

### 3.3. Nutritional Status, Health, and Reproduction-Related Characteristics of Mothers

Around two-thirds (66.8%) of the maternal BMI were within the recommended range (BMI = 18.5–24.9 kg/m^2^). But 13.4% of mothers had BMI less than 18.5 kg/m^2^. Regarding their antenatal care (ANC) utilization, 85.9% of them had four and above visits during the pregnancy of the selected children. 458 (64.8%) of the mothers were receiving information about infant and child feeding during their ANC visits.

### 3.4. Nutritional Status of Infants and Young Children

**Figure 1:**
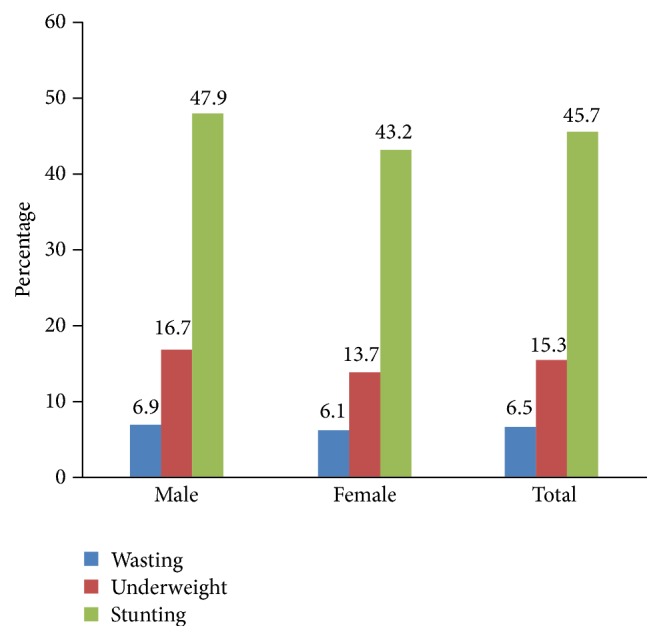
Prevalence of undernutrition by sex of children in Gondar Town, northwest Ethiopia, 2014.

The overall prevalence of stunting was 45.7% (95% CI: 41.9, 49.5%), of which 21.2% were severely stunted, whereas the prevalence of wasting was 6.5% (95% CI: 4.8, 8.2%). Stunting and underweight were higher among females (Figure 1).

### 3.5. Factors Associated with Stunting and Wasting

According to the multivariable binary logistic regression, being female (AOR = 1.65; 95% CI: 1.10, 2.48) and household family size of 4–6 (AOR = 2.48; 95% CI: 1.10, 2.15) were positively associated with stunting. However, initiation of breast-feeding within one hour of birth (AOR = 0.67; 95% CI: 0.46, 0.96) was negatively associated with stunting (Table 4).

Higher odds of wasting were observed among children who were not exclusively breast-fed for the first six months (AOR = 5.63; 95% CI: 1.7, 18.36), faced eating problems in the last two weeks prior to the date of survey (AOR = 2.96; 95% CI: 1.13, 7.78), and had daily laborer fathers (AOR = 2.63; 95% CI: 1.10, 6.27) (Table 5).

## 4. Discussion

Childhood undernutrition was a significant public health problem in the study area. Eating problem, exclusive breast-feeding status for the first six months, family size, and breast-feeding initiation time were associated with undernutrition.

The prevalence of stunting was 45.7% (95% CI: 41.9, 49.5%), which is comparable to the finding of a study done in Dembiya District, Ethiopia (46%) [4], but it is lower than other districts of Ethiopia: Shire (Inda Selassie) (56.6%) [5] and Belesa District (57.7%) [2]. This may be due to Ethiopian recent nutrition programme that gives special attention to child nutrition and the promotion campaign on infant and young child feeding options. In addition, in this study, most of the mothers were educated: attended secondary and above school. Educated mothers can easily understand the effect of undernutrition on child health and survival. Furthermore, educated mothers are more likely to provide nutrient dense, adequate, and frequent complementary feeding to their children. Previous reports have also documented that boosting maternal knowledge is one of the fundamental instruments to break the intergenerational cycle of malnutrition [22].

Furthermore, the observed prevalence of stunting is also higher than what is reported in Indonesia (28.4%) [19], Bhutan (34.9%) [23], and EDHS (40%) [16]. This could be explained by cultural and food security status differences which may have an effect on the infant and young child feeding practices in the three settings: Ethiopia, Indonesia, and Bhutan.

The prevalence of wasting in the study area is in line with the finding from Ethiopia (9%) [24]. However, the prevalence is higher than the reports in Kenya (2.6%) [25] and Nigeria (3.7%) [26]. This is due to low family income in Ethiopia compared to Kenya and Nigeria. Supporting this argument, paternal occupation was one of the determinant factors of infant and young child wasting, according to the finding of this study [27].

In agreement with another study [28], higher odds of stunting were observed among females compared to males. In contrast, stunting was higher among males compared to females in studies done in Indonesia [19] and China [29]. This might be due to cultures in which parents give special attention and feed male children better than females.

Similar to other studies [30–32], the odds of stunting were higher among children from larger families compared to children from a family with less than three members. This is due to the fact that when families are large and their resources are limited, the available food is shared by all members, reducing the amount individuals get. In addition, low-quality foods and inappropriate feeding practice may contribute to the high prevalence of undernutrition [33–35].

Exclusive breast-feeding for the first six months is one of the strategies employed to decrease the burden of undernutrition [30]. Similarly, the higher odds of wasting were observed among children who were not exclusively breast-fed for the first six months compared to their counterparts. This is due to an early and late introduction of complementary foods. Early initiation of complementary feeding has been strongly associated with infection, which leads to an increased energy demand and loss of appetite and nutrients [36]. In addition, after six months, the energy requirements of children are not satisfied with breast milk alone [37].

In this study, the odds of being wasted were nearly 3 times (AOR = 2.96; 95% CI: 1.13, 7.78) higher among children who had eating problems for the last two weeks prior to the survey compared to children who did not have eating problems. This finding is in line with another finding from Ethiopia [38]. It is evident that recurrent infection is one of the determining factors for the high prevalence of undernutrition among under-five children [39].

Higher odds of wasting (AOR = 2.63; 95% CI: 1.10, 6.27) were found among children whose fathers were daily laborers compared to children whose fathers were government employees. This finding is supported by finding from Bangladesh [40]. In Ethiopia, men are the main source of family income. Family income depends on the type of paternal occupation, as the food purchasing power of the family is dependent on family income. Studies show that poor household wealth status has been strongly associated with inadequate intake of nutrient and poor personal and environmental hygiene, which are likely to increase the frequency of infection and undernutrition [29, 41]. In addition, government employees have a fixed monthly income, and most of them are educated. Educated individuals are more likely to follow the recommended child feeding practices, which improve the nutritional status.

## 5. Limitation of the Study

Even though adequate training was given to data collectors and supervisors, instruments were calibrated frequently, and respondents were clearly informed about the objectives of the study; still, there might be some limitations. First, there might be some intraobserver bias during weighing and recording. Second, the study may not show the causal relationship between undernutrition (stunting and wasting) and selected variables due to its cross-sectional nature.

## 6. Conclusion

Despite the fact that Ethiopia has been planning to reach zero level of childhood undernutrition, the study showed a significant public health problem in the study area. The finding alerts the public authorities about the implementation of undernutrition reduction initiatives. Therefore, strengthening early initiation and exclusive breast-feeding, promoting healthcare seeking behavior, and designing social support programme for poor family are recommended to reduce the burden of childhood undernutrition.

## Figures and Tables

**Table 1:** Sociodemographic and socioeconomic characteristics of parents, Gondar Town, northwest Ethiopia (*n* = 707).

Variables	Frequency	Percentage
*Age of mothers (in years)*		
<25	165	23.3
25–30	364	51.5
31–35	102	14.4
>35	76	10.7
*Mothers' marital status*		
Currently married	619	87.6
Currently unmarried^$^	88	12.4
*Mothers' religion *		
Orthodox	501	70.9
Muslim	201	28.4
Others	5	0.7
*Mothers' educational status *		
Unable to read and write	220	31.1
Primary	210	29.7
Secondary and above	277	39.2
*Husbands' education *		
Unable to read and write	85	12
Primary	315	44.5
Secondary and above	307	43.4
*Mothers' occupation *		
Housewife	500	70.7
Government employee	38	5.3
Merchant	52	7.4
Daily laborer	117	16.5
*Husbands' occupation *		
Government employee	233	32.9
Merchant	207	29.3
Daily laborer	184	26.0
Own private work	83	11.7
*Family size *		
≥7	68	9.6
4–6	389	55.0
≤3	250	35.4
*Number of under-five children*		
Two and above	142	20.1
One	565	79.9
*Monthly income *		
<2175 ETB^#^	446	63.1
≥2175 ETB	261	36.9

^$^Single, widowed, or divorced; ^#^Ethiopian Birr (1$ = 26.7 ETB).

**Table 2:** Selected health-related and sociodemographic characteristics of the children aged 6–59 months, Gondar Town, northwest Ethiopia (*n* = 707).

Variables	Frequency	Percentage
*Sex of the child*		
Male	378	53.5
Female	329	46.5
*Age of the child (in months)*		
6–11	74	10.5
12–23	181	25.6
24–35	174	24.6
36–47	155	21.9
48–59	123	17.4
*Birth interval*		
1st child	289	40.9
1 year	28	4.0
2 years	79	11.2
3 years	84	11.9
4 years and above	227	32.1
*Weight changes in the last two weeks*		
Yes	64	9.1
No	643	90.9
*Illness in the last two weeks*		
Yes	152	21.5
No	555	78.5
*Morbidity symptoms *(*n* = 152)^**∗**^		
Diarrhea	83	54.6
Cough	44	28.9
Fever	25	16.5
*Children had eating problem *		
Yes	146	20.7
No	561	79.3
*Types of eating problems *(*n* = 146)^**∗**^		
Loss of appetite	140	89.2
Swallowing problem	2	1.3
Vomiting	15	9.5
*Children had Bitot's spot*		
Yes	4	0.6
No	703	99.4
*Vitamin A supplementation *		
Yes	682	96.5
No	25	3.5

^*∗*^Multiple responses.

**Table 3:** Feeding practices of children aged 6–59 months, Gondar Town, northwest Ethiopia (*n* = 707).

Variables	Frequency	Percentage
*Prelacteal feeding*		
Yes	58	8.2
No	649	91.8
*Initiation of breast-feeding *		
Within one hour	532	75.2
More than one hour	175	24.8
*Children being breast-fed*		
Yes	301	42.6
No	406	57.4
*Exclusively breast-fed *		
No	130	18.4
Yes	577	81.6
*Reasons not to be exclusively breast-fed (130)*		
Work related	16	12.3
Medical problem	4	3.1
Formula feeding has advantage	5	3.8
Maternal perception	25	19.2
Not having enough milk	42	32.3
Lack of family support	27	20.8
Not to breast-feed	11	8.5
*Frequency of complementary feeding *		
Less than 3 times per day	27	3.8
3–5 times per day	541	76.5
More than five times per day	139	19.7
*Material used for complementary feeding *		
Bottle	41	5.8
Cup	46	6.5
Spoon	218	30.8
Fork	14	2.0
Mother hand	137	19.4
His/her hand	430	60.8

**Table 4:** Factors associated with stunting among children aged 6–59 months, Gondar Town, northwest Ethiopia.

Variables	Nutritional status (stunting)		COR (95% CI)	AOR (95% CI)
	Stunted	Not stunted		
*Age of the child*				
6–11	38	36	1.35 (0.76, 2.41)	1.37 (0.85, 1.58)
12–23	80	101	1.02 (0.64, 1.61)	1.00 (0.61, 1.66)
24–35	85	89	1.22 (0.77, 1.94)	1.15 (0.70, 1.89)
36–47	66	89	0.95 (0.59, 1.53)	0.90 (0.54, 1.48)
48–59	54	69	1.00	1.00
*Mothers' educational status*				
Illiterate	95	125	0.92 (0.65, 1.32)	1.04 (0.65, 1.64)
Primary	103	107	1.17 (0.82, 1.68)	1.24 (0.83, 1.84)
Secondary and above	125	152	1.00	1.00
*Husbands' educational status*				
Illiterate	38	47	0.90 (0.56, 1.46)	0.93 (0.51, 1.69)
Primary	140	175	0.89 (0.65, 1.23)	0.88 (0.60, 1.28)
Secondary and above	145	162	1.00	1.00
*Mothers' occupation*				
Housewife	226	274	1.00	1.00
Government employee	17	21	0.98 (0.51, 1.91)	0.91 (0.45, 1.84)
Merchant	24	28	1.04 (0.59, 1.84)	1.04 (0.57, 1.91)
Daily laborer	56	61	1.11 (0.744, 1.67)	1.41 (0.89, 2.25)
*Husbands' occupation*				
Government employee	113	120	1.00	1.00
Merchant	96	111	0.92 (0.63, 1.33)	0.96 (0.64, 1.43)
Daily laborer	75	109	0.73 (0.49, 1.08)	0.68 (0.44, 1.07)
Own private work	39	44	0.94 (0.57, 1.56)	0.95 (0.56, 1.62)
*Family size*				
≥7	27	41	0.88 (0.51, 1.52)	1.10 (0.58, 2.08)
4–6	189	200	1.26 (0.92, 1.74)	**2.48 (1.10, 2.15)** ^*∗∗*^
≤3	107	143	1.00	1.00
*Number of under-five children*				
Two and above	62	80	0.90 (0.62, 1.31)	0.78 (0.51, 1.18)
One	261	304	1.00	1.00
*Sex of the child*				
Male	167	211	1.00	1.00
Female	156	173	1.14 (0.85, 1.53)	**1.65 (1.10, 2.48)** ^*∗∗*^
*BMI of the mothers*				
≤18.4	36	59	1.00	1.00
18.5–24.9	221	251	1.44 (0.92, 2.27)	1.50 (0.94, 2.39)
25–29.9	54	61	1.45 (0.83, 2.52)	1.47 (0.82, 2.62)
≥30	12	13	1.51 (0.62, 3.67)	1.39 (0.55, 3.54)
*Number of ANC visits*				
1–3 times	43	57	0.88 (0.58, 1.35)	0.91 (0.57, 1.45)
4 times and above	280	327	1.00	1.00
*Prelacteal feeding*				
Yes	27	31	1.04 (0.61, 1.78)	0.96 (0.48, 1.94)
No	296	353	1.00	1.00
*Exclusive breast-feeding for the first six months*				
No	61	69	1.06 (0.726, 1.56)	1.11 (0.68, 1.83)
Yes	262	315	1.00	1.00
*Breast-feeding initiation*				
Within 1 hour of birth	232	300	0.71 (0.51, 1.01)	**0.67 (0.46, 0.96)** ^*∗∗*^
More than 1 hour of birth	91	84	1.00	1.00

*∗∗* indicates significance at *p* value less than 0.05.

**Table 5:** Factors associated with wasting among children aged 6–59 months, Gondar Town, northwest Ethiopia.

Variables	Nutritional status (wasting)		COR (95% CI)	AOR (95% CI)
	Wasted	Normal		
*Age of the child *				
6–11	6	68	1.12 (0.38, 3.28)	2.38 (0.47, 12.0)
12–23	8	173	0.58 (0.22, 1.56)	0.87 (0.20, 3.63)
24–35	11	163	0.86 (0.34, 2.13)	0.76 (0.19, 1.09)
36–47	12	143	1.06 (0.43, 2.61)	2.56 (0.69, 9.41)
48–59	9	114	1.00	1.00
*Mothers' educational status *				
Illiterate	14	206	1.11 (0.53, 2.32)	1.04 (0.26, 4.08)
Primary	16	194	1.35 (0.66, 2.76)	1.32 (0.38, 4.62)
Secondary and above	16	261	1.00	1.00
*Husbands' education *				
Illiterate	5	80	0.77 (0.28, 2.09)	1.04 (0.22, 4.97)
Primary	18	297	0.75 (0.39, 1.42)	1.08 (0.38, 3.08)
Secondary and above	23	284	1.00	1.00
*Husbands' occupation *				
Government employee	13	220	1.00	1.00
Merchant	11	196	0.95 (0.42, 2.17)	1.12 (0.46, 2.71)
Daily laborer	18	166	1.84 (0.87, 3.85)	**2.63 (1.10, 6.27)** ^*∗∗*^
Own private work	4	79	0.86 (0.27, 2.71)	0.97 (0.29, 3.29)
*Family size *				
≥7	5	63	0.87 (0.32, 2.35)	0.69 (0.14, 3.48)
4–6	25	364	0.86 (0.30, 2.44)	0.44 (0.13, 1.57)
≤3	16	234	1.00	1.00
*Number of under-five children*				
Two and above	10	132	1.11 (0.54, 2.30)	1.16 (0.39, 3.45)
One	36	529	1.00	1.00
*Sex of the child*				
Male	26	352	1.00	1.00
Female	20	309	0.88 (0.48, 1.60)	0.67 (0.28, 1.58)
*Number of ANC visits *				
1–3 times	8	92	1.30 (0.59, 2.88)	0.95 (0.26, 3.38)
4 times and above	38	569	1.00	1.00
*Exclusive breast-feeding for the first six months*				
No	12	118	1.62 (0.82, 3.23)	**5.63 (1.7, 18.36)** ^*∗∗*^
Yes	34	543	1.00	1.00
*Had eating problem *				
Yes	13	133	1.56 (0.80, 3.05)	**2.96 (1.13, 7.78)** ^*∗∗*^
No	33	528	1.00	1.00

*∗∗* indicates significance at *p* value less than 0.05.

## Abbreviations

ANC: Antenatal care
AOR: Adjusted Odds Ratio
BMI: Body mass index
CI: Confidence Interval
COR: Crude Odds Ratio
EDHS: Ethiopia Demography and Health Survey
ETB: Ethiopian Birr
GDP: Gross domestic product
HAZ: Height-for-Age
IQR: Interquartile Range
MI: Micronutrient Initiatives
SD: Standard deviation
UNICEF: United Nations Children's Fund
WAZ: Weight-for-Age
WHO: World Health Organization
WHZ: Weight-for-Height.

## Data Access

Data will be available upon request from the corresponding author.

## Supplementary Materials

Supplementary Materials The dataset, “mat.5367070.v2”, provided as supplementary material includes the anthropometric data of children aged 6–59 months who participated in the study in MS Excel Workbook format (.xlsx).
